# Motor Imagery in Clinical Disorders: Importance and Implications

**DOI:** 10.3389/fpsyt.2015.00023

**Published:** 2015-02-18

**Authors:** Aidan Moran, Jessica Bramham, Christian Collet, Aymeric Guillot, Tadhg Eoghan MacIntyre

**Affiliations:** ^1^School of Psychology, University College Dublin, Dublin, Ireland; ^2^Centre de Recherche et d’Innovation sur le Sport, Université Claude Bernard Lyon 1, Villeurbanne, France; ^3^Institut Universitaire de France, Paris, France; ^4^Department of Physical Education and Sport Sciences, University of Limerick, Limerick, Ireland

**Keywords:** motor imagery, mental imagery, post-traumatic stress disorder, personality disorders, social anxiety disorder

One of our most remarkable mental capacities is the ability to use our imagination voluntarily to mimic or simulate sensations, actions, and other experiences. For example, we can “see” things in our mind’s eye, “hear” sounds in our mind’s ear, and imagine motor experiences like running away from, or perhaps “freezing” in the face of, danger. Since the early 1900s (1), researchers have investigated “mental imagery” or the multimodal cognitive simulation process by which we represent perceptual information in our minds in the absence of sensory input (2).

Although visual imagery has attracted most research attention to date (3), there has been an upsurge of interest in cognitive neuroscience and sport psychology in non-visual simulation processes such as “motor imagery” (MI) - or the mental rehearsal of actions without engaging in the physical movements involved (4). This trend is attributable mainly to the discovery of close parallels between the neurocognitive mechanisms underlying imagination and motor control. Specifically, inspired by Jeannerod’s (5–7) simulation theory of action representation, researchers have discovered that MI recruits similar neural pathways and mechanisms to those involved in actual movements. For example, Hétu et al. (8) showed that the neural network of MI includes several cortical regions known to underlie actual motor execution. Building on this apparent functional equivalence between imagined and executed actions, the present article explores the implications of research on MI for increased understanding of three clinical conditions – post-traumatic stress disorder (PTSD), personality disorder, and social anxiety disorder (SAD). Before we begin, however, some background information on imagery processes in psychopathology is required.

Arising from Kosslyn’s proposition that mental imagery plays “a special role in representing emotionally charged material” [(9), p. 405; see also Ref. (10)], researchers have examined the role of imagery processes in the onset, maintenance, and treatment of various psychological disorders (11–13). A consistent finding is that negative, vivid, and distressing *involuntary* (“intrusive”) imagery is a “transdiagnostic” feature of depression (14), SAD (15), PTSD (16), and obsessive-compulsive disorder [OCD; (17)]. For example, Weßlau and Steil (14) reported that more than one in three depressed people suffer from involuntary negative mental imagery. Furthermore, people’s capacity to use imagery prospectively is significantly impaired in certain clinical disorders. Thus, Morina et al. (18) discovered that depressed patients were less capable of imagining positive future outcomes than were non-depressed controls. Imagery processes also help in the treatment of psychopathology. Indeed, Holmes et al. (19) evaluated the therapeutic value of “imagery rescripting” [where distressing images are modified to change their associated thoughts, feelings, and behavior; (20)] in the treatment of PTSD. Clearly, imagery research represents “a new and important arena” [Pearson et al. (13), p. 3] for clinical psychology.

Despite increased awareness of imagery processes in psychopathology, there is at least one significant gap in research in this field. Specifically, little is known about the role of MI in clinical disorders. Curiously, despite the multimodal nature of imagery (21), clinical researchers have tended to focus mainly on its *visual* component. Thus, Weßlau and Steil (14) proclaimed that in imagery, although “other sensory components such as smells, sounds, or haptic sensations … may be present … *the visual aspect is the necessary and sufficient condition*” (our italics, p. 274). This proposition may be challenged, however, by evidence that mildly to moderately depressed patients experience proportionately more *somatic* (39.6%) than visual (27.2%) imagery (17). More importantly, MI processes may help to elucidate the mechanisms underlying clinical conditions with distinctive motor components. For example, Chen et al. (22) discovered that depressed patients have difficulties in the mental rotation of hand stimuli. These imagery deficits reflect “an underlying slowing down of motor preparation, which may contribute to psychomotor retardation” (p. 341).

Let us now consider three specific disorders in which MI processes are potentially significant - PTSD, personality disorders, and SAD.

## Post-Traumatic Stress Disorder

Post-traumatic stress disorder typically involves a threat to an individual’s physical integrity [DSM-V; (23)]. This threat may prompt movement execution either through resistance to attack (fight) or through intended escape (flight) (24). Accordingly, it seems plausible that *re-experiencing* a traumatic event in the form of “flashbacks” will involve MI. Corroborating this hypothesis, research shows that flashbacks are associated with increases in various types of motor behavior (25). More recently, neuroimaging paradigms in which individuals with PTSD imagine their traumatic experience or simulate flashbacks have shown increased cerebral blood flow to the motor cortex including the precentral gyrus and supplementary motor area (26, 27). These findings shed light on the neurocognitive mechanisms underlying PTSD disorders because they confirm the involvement of motor cortex in the simulated re-experiencing of traumatic events.

Another link between PTSD and MI processes has emerged from recent studies of the “freeze” response or tonic immobility. Briefly, tonic immobility is an involuntary, reflexive state, characterized by apparent physical paralysis, muscular rigidity, and inability to vocalize (28, 29). For animals, it may be a last line of defense because it reduces the likelihood that predators will continue to attack them (30). The freeze response is more complex in humans, however, as it may be triggered by symbolic events such as the perception that a situation is inescapable (31). Interestingly, although “freezing” was first noted as a characteristic of sexual assault (32) - with up to 37-52% of such assault survivors reporting tonic immobility - it has also been identified among victims of other traumas including physical assault and natural disasters (33). Accordingly, tonic immobility has been proposed as a core sign of trauma in PTSD (34). Unfortunately, peri-traumatic tonic immobility has been shown to predict a poor response to pharmacological treatment (35, 36) - which suggests that psychological processes may be especially significant in this form of PTSD. Recently, Bovin et al. (37) discovered that guilt (i.e., negative evaluation of an action or inaction) mediated the association between tonic immobility and PTSD symptom severity. These authors speculated that guilt may be a mechanism through which individuals develop PTSD following tonic immobility. The argument here is that during the tonic immobility experienced in the trauma situation, victims may feel guilty about their lack of action – which renders them especially vulnerable to developing PTSD. As tonic immobilization is a key risk factor for PTSD, interventions that are targeted to remediate the impact of the freeze response could provide a fruitful strategy for the reduction or prevention of PTSD symptoms (36). Therefore, we propose that rescripting based on MI (“remobilizing”) could prove valuable as an intervention technique for PTSD (38).

Recent studies show that tonic immobility during childhood sexual abuse is associated with the onset of subsequent PTSD symptomatology in adulthood (39). The freeze response, or “learned helplessness,” is especially likely in cases of trauma experienced by infants or young children who are physically unable to escape (40). Further insights into MI processes in PTSD spring from research on the differences between patients’ memories of traumatic events and those of non-traumatic events. Thus, van der Kolk and Fisler (41) suggested that trauma is initially represented using somatosensory information - with traumatic experiences being remembered as bodily sensations. Consistent with this proposal, Malmo and Suzuki Laidlaw (42) found that people who had no memory of childhood sexual abuse prior to therapy were “more kinesthetic than visual” in their orientation to the world. Remarkably, during therapy, the “no memory of trauma” participants became aware of their traumatic memories, and were consistently able to report kinesthetic memory details such as their bodily position in relation to that of the perpetrator (42).

## Personality Disorders

The development of personality disorders, particularly borderline personality disorder, has been strongly associated with early trauma and neglect (43–45). Interestingly, certain kinds of imagery rescripting such as re-imagining adverse early childhood events from an adult perspective have been used to treat personality disorders (46). Imagery rescripting was first used by Arntz and Weertman (47) with the primary objective of revising the perceived meaning of events. For example, an image of a childhood memory might be rescripted constructively by imagining an adult entering the scene and intervening in a positive way (e.g., comforting the child concerned). Typically, the rescripting session with the therapist is recorded and the patient then listens to the recording and practices the exercise again at home, where possible using imagery. Later, patients themselves are required to rescript the adverse event. Although imagery rescripting in a promising therapeutic strategy, its efficacy is mediated by many psychological variables. For example, consider the role of “imagery perspective” or the virtual vantage point-of-view adopted by the person imagining [e.g., first-person versus third-person perspective; (48)]. To illustrate, one can “feel” oneself performing an action with one’s body (first-person perspective) or one can “see” oneself or someone else performing that action (third-person perspective). Imagery perspective is important in the treatment of trauma because McIsaac and Eich (49) found that traumatic images retrieved from a third-person perspective were experienced as less emotional than those retrieved from a first-person perspective. Unfortunately, few studies have explored the relative efficacy of different perspectives [which may involve different levels of embodiment (48)] in rescripting imagery interventions.

## Social Anxiety Disorder

Social anxiety disorder is a highly prevalent and disabling condition that involves fear and avoidance of interpersonal interactions, particularly those that involve potential for social evaluation (50). This disorder is typically characterized by vivid visual imagery, particularly that generated from a third-person perspective (51). According to cognitive models of social anxiety [e.g., by Clark and Wells (52)], people with SAD habitually generate negative images from thoughts, feelings, and bodily sensations to create impressions of how they appear to others from a third-person (“observer”) perspective. Intriguingly, Spurr and Stopa discovered that imagery experienced from a third-person perspective is associated with increased negative self-evaluation by comparison with that occurring from a first-person perspective (53).

One strategy for treating social anxiety involves helping patients to restructure their imagery experiences (54). Thus, Wild et al. (55) developed an imagery-based technique to help people to modify traumatic memories. This technique is effective as a brief treatment for social phobia (56). Its use of imagery rescripting is similar to that pioneered by Arntz and Weertman (47) and involves closing one’s eyes, describing recurring images in social situations, and then imagining that the current self is present at the scene and hence, intervening appropriately. This latter imagery clearly has a motor component as it involves re-imagining actions or movements. Accordingly, MI may be helpful for the treatment of SAD because it can orient patients away from the critical self-focused perspective, thereby reducing “egocentric awareness” (57). By contrast, self-focused attention may impair people’s capacity for perspective-taking, thereby maintaining social anxiety (58).

## Conclusion and Future Directions

In this article, we have presented two main arguments concerning imagery processes in psychopathology. Firstly, we postulated that research on MI processes offers intriguing insights into the neurocognitive mechanisms underlying, and psychological treatment of, certain clinical disorders (specifically, PTSD, personality disorders, and SAD). In addition, we proposed that clinical researchers have much to learn from an emerging theoretical theme in cognitive neuroscience – namely, the idea that the brain is a dynamic predictive system (59) which uses *simulation* as a mechanism for integrating the psychological processes of imagination, perception, and action. More immediately, however, several priorities may be identified for future research on MI in clinical disorders. Firstly, greater theoretical and linguistic precision is required in the delineation of different imagery modalities and experiences. For example, some researchers [e.g., Arntz (46)] use the generic term “imagery” to refer to quite different simulation phenomena such as imagining bodily movements and visualizing scenes. Secondly, in assessing problems such as psychomotor retardation, clinical researchers can benefit from the systematic use of objective measures of MI (60) - especially recently developed psychometric tests (61). Finally, on the basis that dynamic mental practice (i.e., imagining a skill while making associated physical movements) can improve skilled performance through enhanced mental representation (62), it seems plausible that dynamic imagery rescripting could enrich therapeutic interventions for patients suffering from certain disorders (e.g., PTSD).

## Conflict of Interest Statement

The authors declare that the research was conducted in the absence of any commercial or financial relationships that could be construed as a potential conflict of interest.
